# Estrogen Hormones’ Implications on the Physiopathology of Temporomandibular Dysfunction

**DOI:** 10.3390/jcm13154406

**Published:** 2024-07-27

**Authors:** Daniel-Corneliu Leucuța, Damaris Anton, Oana Almășan

**Affiliations:** 1 Department of Medical Informatics and Biostatistics, Iuliu Hațieganu University of Medicine and Pharmacy, 400349 Cluj-Napoca, Romania; 2 Department of Prosthetic Dentistry and Dental Materials, Iuliu Hațieganu University of Medicine and Pharmacy, 32 Clinicilor Street, 400006 Cluj-Napoca, Romania

**Keywords:** temporomandibular joint, temporomandibular dysfunction, estrogenic hormones, menstruation disorder, menopause, oral contraceptives, polycystic ovary syndrome

## Abstract

**Background/Objectives:** Temporomandibular dysfunction syndrome consists of several disorders of the masticatory system, namely those of the muscles, the joint itself, as well as the dental and periodontal system. This syndrome is often characterized by pain and an inability to perform functions within the dental–maxillary apparatus, which creates a certain degree of disability in patients. Women are more susceptible to this syndrome than men and hormonal factors, particularly estrogen, are central to its etiology and physiopathology. **Methods:** A comprehensive literature search was conducted using PubMed/MEDLINE, Scopus, Embase, and Web of Science databases regarding articles published from January 2008 to December 2023. Two authors conducted searches in the mentioned databases based on a pre-established search strategy using agreed-upon keywords. Additionally, each review author performed the selection process of eligible studies based on established inclusion criteria. The Newcastle–Ottawa scale and Risk of Bias tool 2 were used to assess each article for its methodological quality. **Results:** Of the 1030 records found in the four bibliographic databases, 22 studies were included in this review. Polymorphism in the alpha estrogen receptor appears to be significantly more prevalent in women with temporomandibular dysfunction, suggesting a genetic predisposition. There is a significant role of estrogen in the physiopathology of TMD-related pain. Women with polycystic ovary syndrome (PCOS) have a significantly higher incidence of TMD, accompanied by elevated inflammatory factors and decreased progesterone levels. In premenopausal women, there is scientific relevance to the association between beta-estradiol levels and TMD development and progression. The effects of estrogen hormones on temporomandibular dysfunction remain highly debated and challenging. **Conclusions:** These findings emphasize the importance of considering hormonal factors, genetic predisposition, and reproductive life stages in understanding and managing temporomandibular dysfunction. Further research is needed to elucidate the specific mechanisms underlying these associations.

## 1. Introduction

Hormonal factors, especially estrogen, are discussed in the etiology and physiopathology of temporomandibular dysfunction [1]. Estrogen plays a vital role in a woman’s life, influencing reproduction and various other aspects of her well-being, such as her psychological health [2]. Disruptions in the monthly menstrual cycle due to modern lifestyles can lead to hormonal imbalances, which occur when this cycle is disrupted due to the tumultuous life that women of the third millennium often lead [3]. All aspects of a woman’s lifestyle, including her diet, physical activity, stress management, professional activities, and adherence to her circadian rhythm, influence her hormonal status [3,4].

The temporomandibular joint (TMJ) serves as the connection between the mandible and the temporal bone of the skull [5]. This joint allows for complex movements essential for chewing and speaking. The anatomy involves various components such as the condyle, glenoid fossa, and articular disc, which work together to facilitate smooth joint function, and also ligaments, which provide stability to the joint, while muscles control its movements [5]. The normal function of the TMJ involves coordinated movements of the condyle and disc consisting of rotation of condyles on their axis and translation of the condyle–disk ensemble during mouth opening and closing [6]. Dysfunction in this joint can lead to various symptoms and requires a comprehensive understanding of its anatomy and function for diagnosis and treatment [6].

As individuals age, certain factors may interfere with normal TMJ function, leading to dysfunction within the structures of the masticatory system, known as etiological factors [7]. There are five major categories of etiological factors implicated in temporomandibular dysfunction: occlusal status, trauma, emotional stress, parafunctions, and pain [7].

In addition to their effect on reproductive life, estrogen hormones have various effects on the temporomandibular joint structures [8]. The effect of estrogen on the temporomandibular joint disc involves reducing the activity and expression of the proteoglycan 4 gene, an essential molecule for joint lubrication [9]. Elevated estrogen levels have also been linked to increased action of collagenase 1 and stromelysin-1 in fibrochondrocytes [10,11]. Some studies have concluded that high levels of estradiol increase the activity of proteases, leading to a reduction in the production of extracellular matrix elements crucial for maintaining the integrity of the joint disc [8]. Increased activity of sodium channels due to estrogen suggests heightened pain perception, potentially favoring the presence of pain associated with temporomandibular dysfunction [12]. Estrogen’s effects result from its binding to two types of receptors, alpha and beta, found in the central and peripheral nervous systems, as well as in the mobilizing muscles of the temporomandibular joint [8]. Increased expression of alpha receptors and decreased expression of beta receptors may promote inflammation [8]. Estrogen is implicated in inflammatory responses in the temporomandibular joint, particularly in modifying pain signals [1]. Regarding the cartilage from the mandibular condyle level, estradiol has been shown to decrease the thickness of the fibrocartilage layer and cellular proliferation [13].

To the best of our knowledge, there has been no research undertaken on the recent existing literature regarding estrogen hormones’ implications on the physiopathology of TMD that involves human subjects only. Therefore, this study aimed to explore the scientific data available regarding the relationship between estrogen hormones and temporomandibular dysfunction. The goal was to identify the effects of estrogen hormones on the structures of the temporomandibular joint in different phases of the menstrual cycle, as well as in women with various menstrual disorders, including the phase of declining ovarian function and menopause. The implications of estrogen receptors in the temporomandibular joint and the connection between the inflammatory process and joint dysfunction will also be evaluated in this review by consulting the existing scientific literature.

## 2. Materials and Methods

The specialized literature search was conducted using the following databases: PubMed/MEDLINE, Scopus, Embase, and Web of Science. The literature search was carried out through institutional access to databases provided by the Library of the “Iuliu Hațieganu” University of Medicine and Pharmacy in Cluj-Napoca, thus enabling access to a wide range of scientific articles.

Two authors (D.L, A.D.) conducted searches in the mentioned databases based on a pre-established search strategy using agreed-upon keywords. The articles’ publishing targeted period was from January 2008 to December 2023. The duplicate elimination and selecting process of relevant studies was performed individually by each review author based on the inclusion criteria using the https://www.rayyan.ai/ platform (accessed on 5 January 2024). Any discrepancies were discussed and resolved collectively by discussion with a third researcher (O.A.) so that only the articles abiding the selection criteria were included in this review. Regarding discrepancies during the selection phase of the articles, if the authors involved in the process had different opinions regarding a study’s inclusion, a discussion was started until they came to a common decision. In screening stages of titles and abstracts, the decision in case of incertitude is to include the article. Then, in the selection phase where the full text of the article is read, the decision to include article becomes a clear one.

The results obtained in each database were selected according to the inclusion and exclusion criteria from the following table (Table 1):

Search strategies from the databases have been undertaken (Table 2):

The total number of articles resulting from the database search was entered into the platform https://www.rayyan.ai/ (accessed on 5 January 2024), where duplicate elimination and selection of relevant studies were conducted. After duplicate articles were removed, the remaining ones were evaluated for eligibility. The two authors individually reviewed the articles’ title and abstract to select relevant studies according to predefined inclusion criteria. Each full-text article was obtained, read, and assessed in depth to determine its inclusion in the review. 

Studies considered to be eligible by both authors, based on the inclusion criteria, were included in the review. 

All eligible studies were thoroughly assessed by each author to extract data such as the year of publication, study design, patient cohort, main results, and conclusions.

The observational studies were assessed for bias using the Newcastle–Ottawa scale (NOS), while the randomized controlled studies were assessed using the Risk of Bias tool 2 (RoB 2) from Cochrane Collaboration. Each article was assessed by two authors (DCL and OA), and discrepancies were solved by discussion. The NOS evaluates studies based on three broad criteria: selection of study groups (examining the adequacy of case definition, representativeness of the cases, selection of controls, and definition of controls), comparability of the groups (on the basis of the design or analysis, ensuring that the studies account for the most important confounding factors), and ascertainment of either the exposure or outcome of interest (evaluates the method of determining the exposure, the outcome, the duration and adequacy of follow-up for cohort studies, or the non-response rate for case–control studies). RoB 2 assesses five domains of bias: bias arising from the randomization process, which evaluates the adequacy of random sequence generation and allocation concealment; bias due to deviations from intended interventions, examining whether participants received the intended interventions and if deviations were addressed; bias due to missing outcome data, assessing the completeness of outcome data; bias in measurement of the outcome, evaluating the appropriateness of the outcome measurement and blinding of outcome assessors; and bias in selection of the reported result, examining if results are selectively reported. Each domain is rated as “low risk of bias”, “some concerns”, or “high risk of bias”, leading to an overall risk of bias judgment for each study. 

## 3. Results

**Figure 1:**
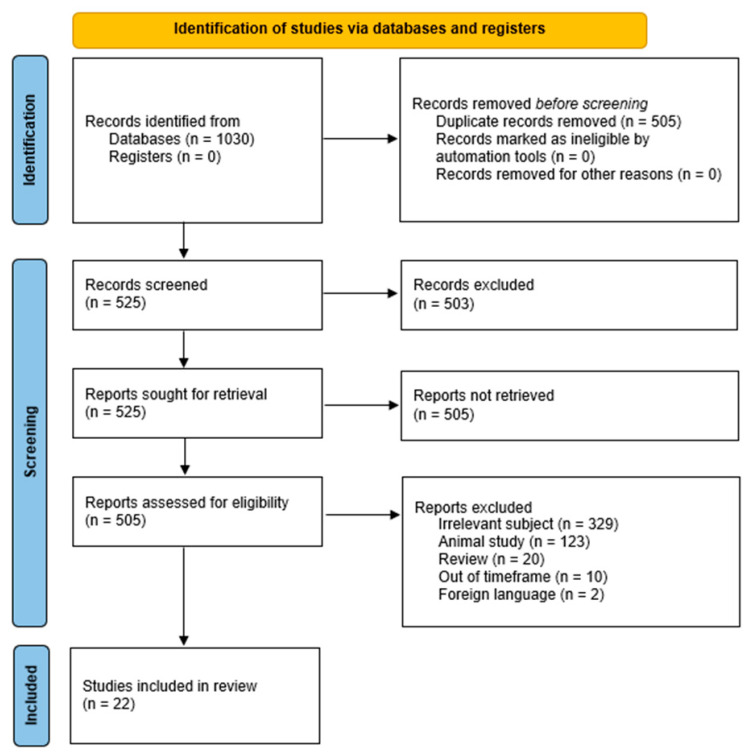
PRISMA 2020 flow diagram for new systematic reviews which included searches of databases and registers only [14] https://www.prisma-statement.org/prisma-2020-flow-diagram (accessed on 15 February 2024).

A total of 1030 records were obtained from the search of the four bibliographic databases (Figure 1). Manual assessment identified 525 duplicate articles that were excluded from the list. The screening of the titles and abstracts eliminated 503 other articles. During the assessment of eligibility, 503 studies were excluded due to being irrelevant to our aims, animal studies, reviews, out of the timeframe, or in foreign languages. Finally, 22 studies were included in our review.

The studies selected for this review have been divided into four categories, which will be discussed as such, to facilitate the understanding of the multiple implications that estrogen hormones have on joint dysfunction pathology (Table 3, Table 4, Table 5 and Table 6).

### Risk of Bias Assessment

Most of the studies were reported as case–control ones. We assessed the observational studies with the Newcastle–Ottawa scale risk of bias assessment tool (Table 7). At least some of the studies were incorrectly labeled as case–control studies, and in reality, they were cross-sectional studies. The definition of cases and controls was adequate for the majority of the studies (19–90%) using RDC/TMD criteria. Only four studies (19%) had a representative sample of cases. The selection of controls was from the same source as that for the cases in 12 studies (57%). A small number of studies (3–14%) used adequate controlling for confounders—one study (5%) using multivariate methods, and two studies (10%) using matching. The assessments of the exposure to estrogen, progesterone levels, or polymorphisms, are unlikely to be subject to bias. The same method of ascertainment was used both for cases and controls in all the studies. Only one study (5%) suffered from some degree of non-response rate.

**Figure 2:**
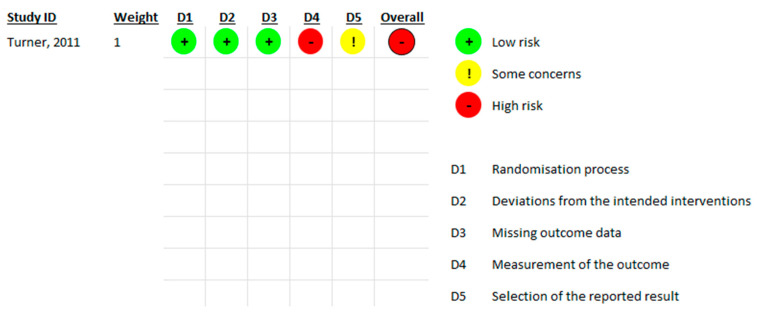
Cochrane Collaboration Risk of Bias tool 2 assessment results [20].

Only one study (Turner, 2011 [20]) was a randomized controlled study. We used the Risk of Bias tool 2 from Cochrane Collaboration to assess the quality of the study. The randomization process, deviations from the intended interventions, and missing outcome data domains were at low risk of bias (Figure 2). There were some concerns of bias regarding selective reporting since no prepublished protocol was available for comparison. There was a high risk or bias for measurement of the outcome, since the patients measured their level of pain, which is a subjective measure.

## 4. Discussion

Five studies compared TMD with healthy patients and found significant differences in estrogen receptor α polymorphisms or receptor expression (Table 3). No significant differences were observed for Catechol-O-Methyl-Transferase [19], nor for tumor necrosis factor α [17] polymorphisms. 

The normalizing of hormonal fluctuations using continuous oral contraceptive therapy was inferior to dental hygienist-delivered pain self-management training for women with TMD pain in a randomized controlled trial [20]. This is in apparent contrast with a case–control study where estrogen levels were associated with a higher level of inflammation [23]. The study assessed the effect of estrogen added in vitro to monocytes sampled from TMD and control participants. Therefore, the authors concluded that a hyperinflammatory pattern caused by estrogen in women with TMD could lead to increased clinical pain, possibly through stimulation of the central nervous system [23]. Maybe the estrogen levels in the study do not replicate the levels found in the randomized controlled trial, and this could explain the absence of efficacy of oral contraceptives. Another discrepancy was observed between the RCT study [20] and a case–control study on oral contraceptives that increased the pain threshold for pressure [25]. However, the case–control study did not find a difference between the reported pain that was assessed in the RCT, and thus the two studies measured different outcomes. Two studies found no difference in estrogen levels in females [21,24]. One study found low levels of serum progesterone associated with TMD in females [21] and one found high levels of testosterone in males associated with idiopathic condylar resorption [24]. One study found that hormonal fluctuations influenced the level of TMJ pain. These findings suggest that estrogen levels may have different effects depending on the sex of the patient. The absence of differences in estrogen levels in several studies might be explained by an unaccounted variability (by matching) in menstrual cycle sampling between the groups. The pain intensity variations during the menstrual cycle might be explained by the independent effect of other hormones, or by interaction between the estrogen effect and that of other hormones. 

Two studies found a significantly higher frequency of TMD in PCOS patients compared to healthy participants [26,28]. The PCOS group had lower mid-luteal progesterone and higher TNF-alpha, matrix metalloproteinase (MMP) 1, 8. No significant differences were found between PCOS with TMD and without TMD concerning estrogen, MMP 1, 8, TNF-alpha, IL-1b [28]. Pregnancy and menstrual disorders are associated with higher frequency of TMD [27,29]. The above-mentioned studies show that several diseases and physiological situations, associated with hormonal changes, are also related to TMD. In the case of PCOS, several hormones were shown to have different levels in TMD and healthy participants. This is the second study [26] to mention low levels of progesterone as another study [21] linked to TMD.

Two studies could not find a significant association between hormone replacement therapy and TMD [30,32]. Three studies found that the presence of TMD was higher in postmenopausal females compared to premenopausal females [33,34,36]. One study did not find a statistically significant difference concerning estrogen levels between postmenopausal females with and without TMD, although the levels were higher in the TMD group [36]. A significantly lower level of estrogen was found in postmenopausal women with TMD compared to those without TMD [31]. Joint degenerative disease is associated with aging, irrespective of the joint, and TMJ makes no difference. The findings of differences between pre and postmenopausal women, can thus be explained, at least in part, by the aging process. Age should be controlled in future studies assessing hormone levels in relation to TMD. The absence of an association between hormone replacement therapy and TMD is an interesting finding. The absence of an association cannot be easily proved, since a *p* value above 0.05 does not exclude an association. It is difficult to correctly assess the level of estrogen hormones due to the menstrual cycle and the multiple factors that influence the menstrual cycle. A hypothesis might be that estrogen hormones might be implicated in the development of the TMJ structures during childhood, which might causally induce the TMD over time. After the development phase of TMJ till adulthood, estrogen’s influence on the joint structure might lessen or disappear. Besides this, estrogen levels might be implicated, regardless of the age in the pain perception in the TMJ. Another confounding factor might be the addressability of the patients suffering from TMD to health services, which might be higher in younger patients compared to older patients. 

In a 2009 case–control study by Dasilva et al. [15], researchers aimed to explore the relationship between polymorphisms in the alpha estrogen receptor gene and the susceptibility to temporomandibular dysfunction (TMD) in women. They analyzed genetic variations of the XbaI/PvuII nucleotide polymorphism in the alpha estrogen receptor gene from DNA samples. Their analysis revealed that the [GC] haplotype at the XbaI site was significantly more prevalent in both TMD groups compared to the control group, indicating an increased predisposition to TMD among women with this haplotype. There were no differences in the [GC] haplotype between symptomatic and asymptomatic TMD groups, suggesting its role solely in TMD pathogenesis rather than pain mechanisms. The study concluded that specific genetic variations in the alpha estrogen receptor gene may elevate the risk of TMD development in women, shedding light on the varying contributions of the estrogen hormone to TMD occurrence between genders and the genetic factors involved in TMD physiopathology.

Another case–control study conducted by Stemig et al. in 2015 [16] evaluated the relationship between the two polymorphisms of the alpha estrogen receptor gene (XbaI and PvuII) and degenerative joint disease. The conclusion of the study was that the presence of polymorphism in the alpha estrogen receptor gene could be implicated in the degenerative effect on the joint bone within degenerative temporomandibular joint disease. However, the authors advocate for further studies involving larger patient cohorts to confirm these results, which, in this case, were not statistically significant. The study’s limitations must be considered for the validity of the results; specifically, the patient sample size with degenerative TMD was too small, as was the number of subjects in the control group. The patient’s age was not taken into account, so the comparison between the two groups cannot be precise, yielding insignificant results. Patient selection was not based on precise clinical and radiological diagnoses, which could affect the accuracy of their selection in the study. It is important to note that further research is needed to fully understand the role of alpha estrogen receptor gene polymorphism in degenerative temporomandibular joint disease.

In 2020, Dalewski et al. [17] published a study aiming to investigate the role of estrogen receptor 1 (ESR1) variant rs1643821 and tumor necrosis factor alpha (TNF-α) variant rs1800629 as potential genetic factors in the occurrence of anterior disc displacement without reduction (ADDwoR) in the temporomandibular joint, specifically exploring the inflammatory mechanism involving these factors. In the odds ratio analysis, a statistically significant association was found between ESR1 rs1643821 and the frequency of anterior disc displacement without reduction. However, the TNF-α polymorphism was statistically insignificant in relation to ADDwoR. In contrast to the results obtained regarding the TNF-α rs1800629 polymorphism, which did not show a statistically significant association with the development of anterior disc displacement with reduction (DDI), Furquim et al. [37] demonstrated that this genetic variant of TNF-α was positively associated with the occurrence of temporomandibular disorders in general. Brazilian patients with TMD were found to have 2.87 times higher odds of having the GA genotype compared to the control group.

Another study in 2021 [18] evaluated the association between the expression of the estrogen receptor alpha 1 gene and temporomandibular joint dysfunction, specifically in samples of articular disc collected from 27 patients selected according to specific criteria. The necessity of researching this association comes from the effect of estrogen hormones on the inflammatory process in the temporomandibular joint by binding to estrogen receptor alpha 1 present in the articular fibrocartilage, specifically triggering the inflammatory cascade through MMP-9 and MMP-13, as concluded in studies on animal models [38]. This study [18] did not find any statistically significant difference in the expression of the estrogen receptor alpha 1 gene concerning condylar fracture, ADDwR, and ADDwoR. However, further studies with larger patient cohorts are needed to confirm these results. 

In a more recent study from 2023, Roudgari et al. [19], found an association between the AA and GA genotypes of ESR1 rs1643821 and an increased likelihood of developing TMD. Additionally, the presence of the TT genotype of ESR2 rs1676303 had a protective effect against the risk of developing TMD. The main conclusion drawn was that ESR1 and ESR2 are involved in the risk of developing TMD.

The study, conducted in 2011 by Judith A. Turner et al. [20], aimed to assess the impact of hormonal fluctuations on temporomandibular disorder pain in women. Three interventions were tested: self-management training by a dental hygienist (SMT), targeted self-management training focused on menstrual cycle changes (TSMT), and continuous oral contraceptive therapy (COCT), aiming to stabilize hormones that could influence TMD pain. Both SMT and TSMT involved in-person sessions and phone calls promoting cognitive–behavioral pain therapies and self-management techniques. COCT involved oral contraceptive therapy. Results showed SMT and TSMT were more effective than COCT at reducing TMD pain symptoms at 12 months and that COCT had adverse effects like irregular bleeding and weight gain. The study did not conclude an increase in the effectiveness of pain self-management therapy based on the menstrual cycle phases.

A 2013 observational study by Madani et al. [21] investigated the link between sex hormone levels and internal TMJ derangements in women with TMD and menstrual cycles. They found that women with TMJ derangements had significantly lower progesterone levels compared to controls, but no difference in 17β-estradiol levels. Moreover, joint crepitus was more frequent in women with lower progesterone levels. This study suggests progesterone may play a larger role than estrogen in TMJ dysfunction. However, limitations include the observational design, small sample size, and focus only on internal TMJ derangements. Additionally, findings from an animal study imply progesterone interacts with estrogen, affecting its effects on pain and inflammation [21,39,40].

In a 2015 observational study by Vilanova et al. [22], the influence of hormonal fluctuations within the menstrual cycle on pain level, maximum occlusal force (MOF), and masticatory performance in women with painful temporomandibular dysfunction was investigated. Previous studies have shown that TMD pain intensity increases during periods of low estrogen levels or sudden decreases, suggesting that pain could be a key factor in masticatory muscle dysfunction [41]. The study [22] compared two groups of patients: one group in which parameters were evaluated based on menstrual cycle phases and another group under oral contraceptive therapy, for which the same parameters were also evaluated. Results showed that TMD-associated pain varies throughout the menstrual cycle, increasing in intensity during the luteal and menstrual phases. No alterations were found in maximum occlusal force and masticatory performance in relation to the menstrual cycle. Therefore, it was concluded that hormonal fluctuations intensify TMD pain without affecting masticatory function.

A 2017 observational case–control study by Dasilva et al. [23] aimed to investigate whether women with TMD exhibit a hyperinflammatory response mediated by monocytes compared to women in a control group, and to examine the association of monocyte-mediated inflammatory response with clinical pain. Previous studies have shown that estrogen hormones have both pro-inflammatory and anti-inflammatory effects on cellular populations; thus, the role of estrogens in monocyte-mediated inflammatory response in TMD is not fully understood [42,43]. The authors found that unstimulated monocytes in TMD patients expressed eight times more IL-6 than those in the control group, and estrogen receptor beta levels were significantly lower in the monocytes from the TMD group, while estrogen receptor alpha levels did not differ. Inhibiting estrogen receptor alpha did not affect the toll-like receptor 4 (TLR4) response, suggesting its minor role in TMD’s hyperinflammatory response. Additionally, TMD women showed a systemic hyperinflammatory response with increased cytokine release from monocytes, enhanced by estrogen. The study concludes that estrogen contributes to TMD-associated pain, with increased cytokine release exacerbating pain, possibly via central sensitization. Higher IL-6 production in patients with greater pain sensitivity suggests its potential association with TMD severity. Reduced estrogen receptor beta expression in TMD monocytes may contribute to TMD’s hyperinflammatory phenotype, while estrogen receptor alpha is not involved [23].

A more recent study from 2021 by Yuan et al. [24] evaluated the contribution of sex hormones in idiopathic condylar resorption (ICR), an aggressive form of TMJ disorder that often occurs in adolescents around the pubertal growth spurt. Their study aimed to investigate the role of serum sex hormone levels in the pathogenesis of ICR, particularly focusing on the controversial role of estrogen imbalance as a potential etiological factor. The study results revealed that women with ICR did not have different serum estrogen levels compared to the disc displacement (DD) group. However, men with ICR showed increased serum testosterone levels compared to patients with DD. They also had relatively higher estrogen levels, although the difference was not statistically significant. The study concluded that low estrogen levels did not contribute to ICR, but testosterone imbalance in men was associated with ICR. 

Vignolo et al. study [25] analyzed the influence of the menstrual cycle and oral contraceptives on the painful pressure threshold of masticatory muscles in patients with myofascial masticatory pain (MFP). The authors found that the use of oral contraceptives can modulate the painful perception in these patients with MFP. 

Sidika et al. study [26] on PCOS and TMD showed a significantly higher incidence of TMD in women with polycystic ovary syndrome (PCOS) compared to the control group. It was suggested that increased activity of matrix metalloproteinases (MMPs) in patients with PCOS with degenerative changes in the TMJ was leading to tissue destruction within the inflammatory response, which could contribute to the increased incidence and severity of temporomandibular dysfunction in this menstrual disorder. 

Grazia et al.’s study [27] on TMD and pregnancy evaluated the prevalence of TMD in pregnant women and found a significant association between increased estrogen levels during pregnancy and an increased susceptibility to developing TMD.

Yazici et al.’s study [28] focused on the relationship between PCOS and TMD, highlighting significant differences in cytokine levels and matrix metalloproteinases in patients with PCOS compared to those without this syndrome. TNF-alpha was found to be higher in patients with PCOS compared to the control group, as were MMP-1 and MMP-8. Additionally, progesterone levels were lower in the PCOS group compared to the control group, suggesting that fluctuations in this hormone could influence the occurrence of TMD in patients with PCOS. The authors also suggested that chronic inflammation could play a role in the etiology of TMD in women with PCOS. There was a significant relationship between polycystic ovary syndrome and temporomandibular dysfunction through the involvement of inflammatory mediators, as well as the potential role of progesterone, providing a better understanding of the physiopathology of TMD in women with PCOS.

Jedynak et al.’s study [29] investigated the form and frequency of TMD in women with menstrual disorders, highlighting the high prevalence of TMD in this group. The results show that TMD was more frequently present in patients with menstrual disorders, specifically with a prevalence of 92.3%. The most common form of TMD in these patients was internal derangement, reported in 68% of cases. Other relevant results included pain in the masticatory muscles (44.62%) and degenerative joint disease (12.3%). Furthermore, the study found a significant association between hypogonadotropic hypogonadism and dysfunction of the masticatory organ in terms of internal derangements and degenerative joint disease, and women with disorders of the hypothalamic–pituitary axis were more predisposed to TMD.

Nekora-Azak et al.’s study [30] investigated the association between hormonal replacement therapy (HRT) in postmenopausal women and signs and symptoms of temporomandibular dysfunction. There was no significant difference in reported symptoms between the two studied groups. The prevalence of morning headaches was 60% in the entire group, with 40.6% reporting oral parafunctions, without significant differences between the HRT and non-HRT groups. No differences were observed in joint and muscle pain between the two groups, but the joint sounds and jaw deviation during movement were slightly higher in the HRT group, although not significantly. The study concluded that there was no significant impact of HRT on TMD signs and symptoms. Further research is needed to evaluate other potential factors contributing to TMD development and management in postmenopausal women. 

An observational study from 2014 [31] explored the possible role of estrogen hormones in temporomandibular dysfunction. A significant statistical association between beta-estradiol levels and TMD was found, as well as one between age and estrogen levels in the TMD group. All results indicated the role of female reproductive hormones, especially estrogen, in the etiology of TMD, particularly in the development and progression of TMD in reproductive-aged women. 

The observational study from 2016 [32] evaluated the prevalence of TMD in postmenopausal women; the relationship between TMD, pain, and hormone replacement therapy; and the role of hormones in TMD physiopathology. The study concluded that hormone replacement therapy does not influence TMD pain in postmenopausal women. The mechanism by which hormones influence TMD pain remains unclear and requires further study.

Another observational study from 2018 [33] compared the prevalence and severity of TMD between menopausal and premenopausal women. The authors found significantly higher prevalence and severity of TMD in menopausal women compared to premenopausal women, suggesting that hormonal changes during menopause may contribute to the increased prevalence and severity of TMD. Following this study, in 2019, another observational study, conducted by Babouei et al. [34] on the same group of patients, evaluated the relationship between menopause and the prevalence of clinical symptoms of TMD. Among the symptoms examined, the most common was functional incompatibility of the TMJ components. The prevalence of TMD symptoms in premenopausal women included pain during jaw movements, pain in the TMJ and in the masticatory muscles, and partial dysfunction of jaw and joint movements. Reduced estrogen levels during menopause were associated with an accentuated inflammatory background, which increases the incidence and severity of TMD symptoms.

Yosaphat et al.’s study from 2020 [35] examined the effect of estradiol levels on anterior disc displacement in menopausal women. They found higher estradiol levels in women with disc displacement compared to those without. Although not statistically significant, this research suggested an influence of estradiol hormone on the incidence of anterior disc displacement.

The last study under discussion, conducted by Mursu et al. in 2022 [36], aimed to examine the association between the decline phase of ovarian function in women at the age of 46 and temporomandibular dysfunction. The decline phase of ovarian function corresponds to the transition phase of a woman towards menopause, diagnosed based on the value of FSH > 25 IU/l (follicle-stimulating hormone) and the presence of amenorrhea for more than four consecutive months. Two groups were compared: one with women in the decline phase of ovarian function and another group with women before this ovarian decline. In this study, reported pain from TMD, clinical signs of TMD, and the diagnosis of dysfunction were evaluated. Women at the age of 46 in the ovarian decline phase showed a higher prevalence of pain upon palpation of the TMJ, incidence of joint crepitus, and degenerative joint disease compared to women in the pre-decline phase. These differences remained statistically significant even after considering other predisposing factors such as body mass index (BMI), smoking, and parity. These results suggest that the decline phase of ovarian function, corresponding to the transition to menopause, may contribute to the development of TMD in women, leading to increased pain and clinical signs of TMJ degeneration.

There are several common limitations observed in the articles found in our review. All of them are observational in nature except one, which is a randomized controlled trial. Observational studies cannot sustain causality assertions. Most of the studies were case–control ones, a design that comes with frequent selection bias due to the difficulty in identifying a control group arising from the same source as the case group. Nevertheless, we consider that at least some of the studies were incorrectly labeled as case–control, and in reality, they were cross-sectional studies that are less prone to this type of selection bias. A small number of studies used multivariate methods and matching; thus, the control of confounding variables remains an important issue. The assessments of exposure, like the exposure to estrogen, progesterone levels, or polymorphisms, are unlikely to be subject to bias. Also, the assessment of TMD was less likely to be subject to bias to bias, since good diagnostic standards were involved in this diagnosis. The above-mentioned limitations imply that clear causal inferences are difficult to sustain, and that the effects of estrogen hormones on temporomandibular dysfunction remain highly debated and challenging. 

This review has several strengths. It follows a comprehensive and systematic literature search across multiple (five) reputable databases, coupled with a rigorous study selection process. The clear inclusion criteria and detailed assessment of each study ensure relevance and quality. Thorough documentation and transparent methodology further show the review’s credibility, making it a robust and valuable resource for understanding the implications of estrogen hormones on temporomandibular dysfunction.

## 5. Conclusions

TMD sufferers had considerably distinctive estrogen receptor polymorphisms in comparison to healthy subjects. Continuous oral contraceptive therapy was found to be less successful than pain self-management training, indicating that TMD discomfort may not always be relieved by hormone therapies. Hormonal variations, notably estrogen, appear to influence pain differently in men and women, and can fluctuate with the menstrual cycle. Further evidence of a complex relationship between hormonal changes and TMD comes from the association of greater TMD frequency with illnesses like menopausal status and PCOS, which alter hormone levels. There was an association between estrogen hormones and TMD in women based on different stages of reproductive age and menopause and postmenopause. The results point to a challenging interplay between hormone levels and TMD, requiring additional research that considers age, menstrual cycle, and other confounding variables.

## Figures and Tables

**Table 1:** Inclusion and exclusion criteria.

Inclusion Criteria	Exclusion Criteria
Relevant topic studies	Studies published outside the 2008–2023 timeframe
Articles published within the last 15 years	
Articles published in English	Articles published in other languages
Full-text format	Review articles, systematic reviews
Studies involving human subjects	Studies involving animals

**Table 2:** Search strategies for each database.

Database	Strategy	Results
PubMed	(“temporomandibular joint” OR “temporomandibular joint disease” OR “temporomandibular joint disk” OR “temporomandibular joint disorder” OR “temporomandibular joint syndrome” OR “TMJ syndrome” OR TMJ OR “Myofascial pain dysfunction syndrome” OR “TMJ disease” OR “TMJ disorder” OR “tmj disorder” OR tmj OR “tmj disease” OR “tmj syndrome” OR “muscle disorder” OR “temporomandibular articular disk” OR TMD OR “temporomandibular pain”) AND (estrogens OR “estrogen replacement therapy” OR “estrogenic hormones” OR “gonadal hormones” OR “sex hormones “ OR “polycystic ovary syndrome” OR “polycystic ovarian syndrome” OR “menstrual cycle” OR “menstruation disorder” OR “menstruation disturbance” OR postmenopause OR “hormonal oral contraceptive” OR “premenstrual syndrome” OR estradiol OR “estradiol levels” OR progesterone)	368
Scopus	TITLE-ABS-KEY (“TMD” OR “temporomandibular joint” OR “temporomandibular joint disorder”) AND (“estrogens”)	260
Embase	(‘tmd’ OR ‘temporomandibular joint’ OR ‘temporomandibular joint disorder’) AND (‘estrogens’ OR ‘gonadal hormones’ OR ‘sex hormones’ OR ‘menstrual disorders’ OR ‘postmenopause’ OR ‘oral contraceptive agent’)	200
Web of Science	(TS = (temporomandibular joint disorder) OR TS = (TMD) OR TS = (internal derangement)) AND (TS = (estrogens) OR TS = (gonadal hormones) OR TS = (sexual hormones) OR TS = (menstruation disorder) OR TS = (menstrual cycle))	202

**Table 3:** Studies assessing the implications of estrogen receptors from the temporomandibular joint in temporomandibular disorders.

Reference	Year	Study Design and Subjects	Conclusion
Estrogen Receptor-α Polymorphisms and Predisposition to TMJ Disorder [15]	2009	Case–control 200 patients with TMD 100 healthy patients	The polymorphism of the estrogen receptor may play a role in the onset of temporomandibular disorders in women.
Estrogen receptor-alpha polymorphism in patients with and without degenerative disease of the temporomandibular joint [16]	2015	Case–control DNA samples from 42 patients with articular degenerative disease and 36 healthy patients	The presence of polymorphism in the estrogen receptor alpha gene could be involved in the degenerative effect on the joint bone within degenerative temporomandibular joint disease.
Association of Estrogen Receptor 1 and Tumor Necrosis Factor α Polymorphisms with Temporo-mandibular Joint Anterior Disc Displacement without Reduction [17]	2020	Case–control 124 patients with ADD (anterior disc displacement) 126 healthy patients	ESR1 could be involved in the pathogenesis of synovitis and degenerative pathology of temporomandibular joint cartilage and bone
Association of estrogen receptor alpha 1 and TMJ dysfunction: A pilot study [18]	2021	Pilot study 40 articular disc specimens from 27 patients suffering from ADDwRand ADDwoR (anterior disc displacement with and without reduction)	There is no association between the expression of the estrogen receptor alpha gene and age in patients with condylar fracture, ADDwR and ADDwoR
Association of Catechol-O-Methyl-Transferase and Estrogen Receptors polymorphism with Severity of Temporo-mandibular Disorder in Iranian Patients [19]	2023	Case–control Blood samples taken from 100 TMD patients and 103 healthy patients	The ESR1 and ESR2 genes are involved in the risk of developing temporomandibular disorders. The relationship between COMT and TMD was not statistically significant.

**Table 4:** Studies addressing the relationship between estrogen hormone levels and TMD.

Reference	Year	Study Design and Subjects	Conclusion
Targeting Temporo-mandibular Disorder Pain Treatment to Hormonal Fluctuations: A Randomized Clinical Trial [20]	2011	Randomized clinical trial 147 female participants	Long-term benefits of self-applied therapeutic interventions for TMD pain by patients at the recommendation of the dental hygienist have been observed.
A cross-sectional study of the relationship between serum sexual hormone levels and internal derangement of temporomandibular joint [21]	2013	Cross-sectional 47 patients with ADDwR 95 patients without TMD	Low levels of serum progesterone could be associated with internal derangements of the temporomandibular joint. No difference was found for estrogen serum levels.
Hormonal Fluctuations Intensify Temporo-mandibular Disorder Pain Without Impairing Masticatory Function [22]	2015	Case–control 1 group (n = 25) evaluated for menstrual cycle and 1 group (n = 25) evaluated for OC (oral contraceptives) intake	The level of temporomandibular joint pain in women is influenced by hormonal fluctuations (lower pain level in ovulatory phase). The menstrual cycle does not interfere with masticatory function.
Estrogen-Induced Monocytic Response Correlates with Temporo-mandibular Disorder Pain: A Case Control Study [23]	2017	Case–control Blood samples taken from 18 patients (9 with TMD, and 9 controls)	Women with TMD had a systemic hyperinflammatory phenotype, as seen by increased monocytic cytokine release following an inflammatory insult, amplified by estrogen. Monocytes from participants who reported more pain on the VAS scale produced higher levels of IL6 than those from people who indicated lesser pain sensitivity.
Do sex hormone imbalances contribute to idiopathic condylar resorption? [24]	2021	Case–control 94 patients with ICR (idiopathic condylar resorption) 324 patients with DD (disc displacement)	There is no contribution of low estrogen levels in ICR in females, but higher testosterone and estrogen levels in men is related to ICR.
Influence of the menstrual cycle on the pressure pain threshold of masticatory muscles in patients with masticatory myofascial pain [25]	2008	Case–control 36 patients divided into 4 groups: 7 with TMD and no OC intake, 8 with TMD and OC intake, 13 healthy with no OC intake, 8 healthy with OC intake	The menstrual cycle phases do not have a significant impact on the masticatory muscle tenderness, but reported pain is slightly increased during the menstrual phase. The use of oral contraceptives is associated with an increased pain threshold to pressure.

**Table 5:** Studies correlating TMD with various physiological and pathological conditions of the female reproductive system.

Reference	Year	Study Design and Subjects	Conclusion
Is the incidence of temporo-mandibular disorder increased in polycystic ovary syndrome? [26]	2014	Case–control 100 premenopausal women divided into 1 group with PCOS (polycystic ovary syndrome) and 1 healthy group	The incidence and severity of TMD are higher in patients with polycystic ovary syndrome compared to asymptomatic patients.
Cranio-mandibular Disorders in Pregnant Women: An Epidemiological Survey [27]	2020	Case–control 108 pregnant women 90 control women	Pregnant women are more susceptible to TMD
The novel relationship between polycystic ovary syndrome and temporomandibular joint disorders [28]	2020	Case–control 45 PCOS patients 30 healthy patients	There is a significant relationship between PCOS and TMD. The PCOS group had lower mid-luteal progesterone and higher TNF-alpha, matrix metalloproteinase (MMP) 1, 8. No significant differences were found between PCOS with TMD and without TMD concerning estrogen, MMP 1, 8, TNF-alpha, IL-1b.
TMD in Females with Menstrual Disorders [29]	2021	Observational 65 women with menstrual disorders aged between 18 and 40 years and 61 matched by age and gender healthy controls	TMD was more frequent in females with menstrual disorders compared to the control group.

**Table 6:** Studies addressing the connection between TMD and menopause.

Reference	Year	Study Design and Subjects	Conclusion
Estrogen Replacement Therapy Among Postmenopausal Women and Its Effects on Signs and Symptoms of Temporomandibular Disorders [30]	2008	Case–control 91 postmenopausal women with HRT (hormonal replacement therapy) 89 women without HRT	There is no significant association between the use of hormone therapy in postmenopausal women and signs and symptoms of TMD.
Possible role of estrogen in temporomandibular disorders in female subjects: A research study [31]	2014	Case–control 195 patients divided into 2 groups: with TMD and without TMD, and further divided in pre and postmenopausal women.	There is a significant association between estrogen levels and TMD.
Prevalence of temporomandibular disorders in postmenopausal women and relationship with pain and HRT [32]	2016	Case–control 129 TMD patients 155 healthy patients	The use of hormone replacement therapy is not associated with TMD in postmenopausal women. The prevalence of TMD is increased during the reproductive age.
Comparison of temporomandibular disorders between menopausal and non-menopausal women [33]	2018	Case–control 69 premenopausal patients 71 postmenopausal patients	The prevalence of TMD was significantly higher in women in postmenopause compared to those before menopause.
Evaluating the prevalence of temporomandibular joint abnormalities in postmenopausal women [34]	2019	Case–control 69 premenopausal patients 71 postmenopausal patients	Postmenopausal women have a higher prevalence of TMD symptoms.
Effects of estradiol hormone in menopausal women on anterior disc displacement of temporomandibular joint [35]	2020	Case–control 40 postmenopausal women divided into 2 groups: healthy TMJ and ADD	Estradiol influences the incidence of anterior disc displacement, and the average levels of this hormone are higher in menopausal women with anterior disc displacement, but not statistically significant.
Association of climacterium with temporomandibular disorders at the age of 46 years—a cross-sectional study [36]	2022	Cross-sectional 71 climacteric patients 956 preclimacteric patients	The onset of climacteric phase in women is associated with the presence of TMD signs and symptoms.

**Table 7:** Newcastle–Ottawa scale risk of bias assessment for observational studies selected in the review.

Study	Is the Case Definition Adequate?	Representativeness of the Cases	Selection of Controls	Definition of Controls	Comparability of Cases and Controls on the Basis of the Design or Analysis	Ascertainment of Exposure	Same Method of Ascertainment for Cases and Controls	Non-Response Rate
Ribeiro-dasilva, 2009 [15]	*	-	*	*	* (age matched)	*	*	*
Stemig, 2015 [16]	*	-	*	*	-	*	*	*
Dalewski, 2020 [17]	*	-	-	*	-	*	*	*
Doetzer, 2020 [18]	*	-	*	*	-	*	*	*
Roudgari, 2023 [19]	*	-	-	*	- (gender matched)	*	*	*
Madani, 2013 [21]	*	*	*	*	-	*	*	*
Vilanova, 2015 [22]	*	-	*	*	-	*/-	*	*
Ribeiro-Dasilva, 2017 [23]	*	-	-	*	-	*	*	*
Yuan, 2021 [24]	*	*	*	*	-	*	*	*
Vignolo, 2008 [25]	*	-	*	*	-	*	*	*
Soydan, 2014 [26]	*	-	-	*	-	*	*	*
Fichera, 2020 [27]	*	-	*	*	-	*	*	*
Yazici, 2020 [28]	*	-	*	*	-	*	*	*
Jedynak, 2021 [29]	*	-	-	*	* (age, gender matched)	*	*	*
Nekora-Azak, 2008 [30]	*	-	*	*	-	*	*	*
Ahmad, 2014 [31]	-	-	*	-	-	*	*	*
Lora, 2016 [32]	*	-	*	*	-	*	*	*
Farzin, 2018 [33]	-	-	*	-	-	*	*	*
Babouei, 2019 [34]	-	*	*	-	-	*	*	*
Murusu, 2022 [35]	*	*	*	*	* adjusted body mass index (BMI), smoking, parity, and interaction	*	*	-

* Indicates that the criteria is confirmed.

## Abbreviations

Temporomandibular dysfunction (TMD); temporomandibular joint (TMJ); Newcastle–Ottawa Scale (NOS); Revised Cochrane Risk-of-Bias Tool for Randomized Trials (RoB 2); polycystic ovary syndrome (PCOS); anterior disc displacement (ADD); estrogen receptor (ESR); tumor necrosis factor alpha (TNF-α); oral contraceptives (OC); matrix metalloproteinase (MMP); hormonal replacement therapy (HRT); idiopathic condylar resorption (ICR); self-management training (SMT); targeted self-management training (TSMT); continuous oral contraceptive therapy (COCT); maximum occlusal force (MOF); research diagnostic criteria for temporomandibular disorders (RDC/TMD); follicle-stimulating hormone (FSH); body mass index (BMI); myofascial masticatory pain (MFP); visual analog scale (VAS).

## References

[1] Berger M., Szalewski L., Bakalczuk M., Bakalczuk G., Bakalczuk S., Szkutnik J. (2015). Association between estrogen levels and temporomandibular disorders: A systematic literature review. Prz. Menopauzalny.

[2] Navarro-Pardo E., Holland C.A., Cano A. (2018). Sex Hormones and Healthy Psychological Aging in Women. Front. Aging Neurosci..

[3] MacKendrick N.A., Troxel H. (2022). Like a finely-oiled machine: Self-help and the elusive goal of hormone balance. Soc. Sci. Med..

[4] Cameron J.L. (2003). Hormonal Mediation of Physiological and Behavioral Processes That Influence Fertility.

[5] Okeson J.P. (2019). Functional anatomy and biomechanics of the masticatory system. Management of Temporomandibular Disorders and Occlusion.

[6] Dawson P.E. (2007). The temporomandibular joint. Functional Occlusion: From TMJ to Smile Design.

[7] Okeson J.P. (2019). Etiology of functional disturbances in the masticatory system. Management of Temporomandibular Disorders and Occlusion.

[8] Robinson J.L., Johnson P.M., Kister K., Yin M.T., Chen J., Wadhwa S. (2020). Estrogen signaling impacts temporomandibular joint and periodontal disease pathology. Odontology.

[9] McDaniel J.S., Akula Suresh Babu R., Navarro M.M., LeBaron R.G. (2014). Transcriptional regulation of proteoglycan 4 by 17beta-estradiol in immortalized baboon temporomandibular joint disc cells. Eur. J. Oral Sci..

[10] Kapila S., Xie Y. (1998). Targeted induction of collagenase and stromelysin by relaxin in unprimed and beta-estradiol-primed diarthrodial joint fibrocartilaginous cells but not in synoviocytes. Lab. Investig..

[11] Naqvi T., Duong T.T., Hashem G., Shiga M., Zhang Q., Kapila S. (2005). Relaxin’s induction of metalloproteinases is associated with the loss of collagen and glycosaminoglycans in synovial joint fibrocartilaginous explants. Arthritis Res. Ther..

[12] Bi R.Y., Meng Z., Zhang P., Wang X.D., Ding Y., Gan Y.H. (2017). Estradiol upregulates voltage-gated sodium channel 1.7 in trigeminal ganglion contributing to hyperalgesia of inflamed TMJ. PLoS ONE.

[13] Cheng P., Ma X., Xue Y., Li S., Zhang Z. (2003). Effects of estradiol on proliferation and metabolism of rabbit mandibular condylar cartilage cells in vitro. Chin. Med. J..

[14] Haddaway N.R., Page M.J., Pritchard C.C., McGuinness L.A. (2022). PRISMA2020: An R package and Shiny app for producing PRISMA 2020-compliant flow diagrams, with interactivity for optimised digital transparency and Open Synthesis. Campbell Syst. Rev..

[15] Ribeiro-Dasilva M.C., Peres Line S.R., Leme Godoy dos Santos M.C., Arthuri M.T., Hou W., Fillingim R.B., Rizzatti Barbosa C.M. (2009). Estrogen Receptor-α Polymorphisms and Predisposition to TMJ Disorder. J. Pain.

[16] Stemig M., Myers S.L., Kaimal S., Islam M.S. (2015). Estrogen receptor-alpha polymorphism in patients with and without degenerative disease of the temporomandibular joint. Cranio.

[17] Dalewski B., Kamińska A., Białkowska K., Jakubowska A., Sobolewska E. (2020). Association of estrogen receptor 1 and tumor necrosis factor α polymorphisms with temporomandibular joint anterior disc displacement without reduction. Dis. Markers.

[18] Doetzer A.D., Almeida L.E., de Alcântara Camejo F., de Noronha L., Olandoski M., Trevilatto P.C. (2021). Association of estrogen receptor alpha 1 and TMJ dysfunction: A pilot study. Oral Surg. Oral Med. Oral Pathol. Oral Radiol..

[19] Roudgari H., Najafi S., Khalilian S., Ghafarzadeh Z., Hahakzadeh A., Behazin S., Sheykhbahaei N. (2023). Association of Catechol-O-Methyl-Transferase and estrogen receptors polymorphism with severity of Temporomandibular Disorder in Iranian patients. Avicenna J. Med. Biotechnol..

[20] Turner J.A., Mancl L., Huggins K.H., Sherman J.J., Lentz G., LeResche L. (2011). Targeting temporomandibular disorder pain treatment to hormonal fluctuations: A randomized clinical trial. Pain.

[21] Madani A.S., Shamsian A.A., Hedayati-Moghaddam M.R., Fathimoghadam F., Sabooni M.R., Mirmortazavi A., Golmohamadi M. (2013). A cross-sectional study of the relationship between serum sexual hormone levels and internal derangement of temporomandibular joint. J. Oral Rehabil..

[22] Vilanova L.S., Gonçalves T.M., Meirelles L., Garcia R.C. (2015). Hormonal fluctuations intensify temporomandibular disorder pain without impairing masticatory function. Int. J. Prosthodont..

[23] Ribeiro-Dasilva M.C., Fillingim R.B., Wallet S.M. (2017). Estrogen-Induced Monocytic Response Correlates with TMD Pain: A Case Control Study. J. Dent. Res..

[24] Yuan M., Xie Q., Shen P., Yang C. (2021). Do sex hormone imbalances contribute to idiopathic condylar resorption?. Int. J. Oral Maxillofac. Surg..

[25] Vignolo V., Vedolin G.M., de Araujo C., dos R.P., Rodrigues Conti P.C. (2008). Influence of the menstrual cycle on the pressure pain threshold of masticatory muscles in patients with masticatory myofascial pain. Oral Surg. Oral Med. Oral Pathol. Oral Radiol. Endod..

[26] Soydan S.S., Deniz K., Uckan S., Dogruk Unal A., Bascıl Tutuncu N. (2014). Is the incidence of temporomandibular disorder increased in polycystic ovary syndrome?. Br. J. Oral Maxillofac. Surg..

[27] Fichera G., Polizzi A., Scapellato S., Palazzo G., Indelicato F. (2020). Craniomandibular Disorders in Pregnant Women: An Epidemiological Survey. J. Funct. Morphol. Kinesiol..

[28] Yazici H., Islimye Taskin M., Guney G., Hismiogullari A.A., Arslan E., Tulaci K.G. (2021). The novel relationship between polycystic ovary syndrome and temporomandibular joint disorders. J. Stomatol. Oral Maxillofac. Surg..

[29] Jedynak B., Jaworska-Zaremba M., Grzechocińska B., Chmurska M., Janicka J., Kostrzewa-Janicka J. (2021). TMD in Females with Menstrual Disorders. Int. J. Environ. Res. Public Health.

[30] Nekora-Azak A., Evlioglu G., Ceyhan A., Keskin H., Berkman S., Issever H. (2008). Estrogen Replacement Therapy Among Postmenopausal Women and Its Effects on Signs and Symptoms of Temporomandibular Disorders. CRANIO.

[31] Ahmad M., Chalkoo A. (2014). Possible role of estrogen in temporomandibular disorders in female subjects: A research study. J. Indian Acad. Oral Med. Radiol..

[32] Lora V.R.M.M., Canales G.D.l.T., Gonçalves L.M., Meloto C.B., Barbosa C.M.R. (2016). Prevalence of temporomandibular disorders in postmenopausal women and relationship with pain and HRT. Braz. Oral Res..

[33] Farzin M., Taghva M., Babooie M. (2018). Comparison of temporomandibular disorders between menopausal and non-menopausal women. J. Korean Assoc. Oral Maxillofac. Surg..

[34] Babouei M., Farzin M., Vejdani M., Moayedi I. (2019). Evaluating the prevalence of temporomandibular joint abnormalities in postmenopausal women. Rev. Latinoam. Hipertens..

[35] Rosanto Y.B., Rahajoe P.S. (2020). Effects of estradiol hormone in menopausal women on anterior disc displacement of temporomandibular joint. BIO Web Conf..

[36] Mursu E., Yu J., Karjalainen E., Savukoski S., Niinimäki M., Näpänkangas R., Pesonen P., Pirttiniemi P., Raustia A. (2022). Association of climacterium with temporomandibular disorders at the age of 46 years—A cross-sectional study. Acta Odontol. Scand..

[37] Furquim B.D., Flamengui L.M.S.P., Repeke C.E.P., Cavalla F., Garlet G.P., Conti P.C.R. (2016). Influence of TNF-α-308 G/A gene polymorphism on temporomandibular disorder. Am. J. Orthod. Dentofac. Orthop..

[38] Islander U., Erlandsson M.C., Hasséus B., Jonsson C.A., Ohlsson C., Gustafsson J.-Å., Dahlgren U., Carlsten H. (2003). Influence of oestrogen receptor α and β on the immune system in aged female mice. Immunology.

[39] Puri J., Hutchins B., Bellinger L.L., Kramer P.R. (2009). Estrogen and inflammation modulate estrogen receptor alpha expression in specific tissues of the temporomandibular joint. Reprod. Biol. Endocrinol..

[40] Ren K., Wei F., Dubner R., Murphy A., Hoffman G.E. (2000). Progesterone attenuates persistent inflammatory hyperalgesia in female rats: Involvement of spinal NMDA receptor mechanisms. Brain Res..

[41] LeResche L., Mancl L., Sherman J.J., Gandara B., Dworkin S.F. (2003). Changes in temporomandibular pain and other symptoms across the menstrual cycle. Pain.

[42] Vegeto E., Ghisletti S., Meda C., Etteri S., Belcredito S., Maggi A. (2004). Regulation of the lipopolysaccharide signal transduction pathway by 17beta-estradiol in macrophage cells. J. Steroid Biochem. Mol. Biol..

[43] Calippe B., Douin-Echinard V., Delpy L., Laffargue M., Lélu K., Krust A., Pipy B., Bayard F., Arnal J.F., Guéry J.C. (2010). 17Beta-estradiol promotes TLR4-triggered proinflammatory mediator production through direct estrogen receptor alpha signaling in macrophages in vivo. J. Immunol..

